# Ethylenediaminetetraacetic Acid (EDTA) Mitigates the Toxic Effect of Excessive Copper Concentrations on Growth and Gaseous Exchange of *Corchorus capsularis* L. and Improves Copper Accumulation Capabilities

**DOI:** 10.3390/plants9060756

**Published:** 2020-06-16

**Authors:** Muhammad Hamzah Saleem, Shafaqat Ali, Muhammad Kamran, Naeem Iqbal, Muhammad Azeem, Muhammad Tariq Javed, Qasim Ali, Muhammad Zulqurnain Haider, Sana Irshad, Muhammad Rizwan, Saad Alkahtani, Mohamed M. Abdel-Daim

**Affiliations:** 1 MOA Key Laboratory of Crop Ecophysiology and Farming System Core in the Middle Reaches of the Yangtze River, College of Plant Science and Technology, Huazhong Agricultural University, Wuhan 430070, China; 2 Department of Environmental Sciences and Engineering, Government College University, Allama Iqbal Road, Faisalabad 38000, Pakistan; mrizwan@gcuf.edu.pk; 3 Department of Biological Sciences and Technology, China Medical University, Taichung 40402, Taiwan; 4 College of Resources and Environment, Huazhong Agricultural University, Wuhan 430070, China; kamiagrarian763@gmail.com; 5 Department of Botany, Government College University, Faisalabad 38000, Pakistan; drnaeem@gcuf.edu.pk (N.I.); mazeem@gcuf.edu.pk (M.A.); mtariqjaved@gcuf.edu.pk (M.T.J.); drqasimali@gcuf.edu.pk (Q.A.); drmzhaider@gcuf.edu.pk (M.Z.H.); 6 School of Environmental Studies, China University of Geosciences, Wuhan 430070, China; sanairshad55@gmail.com; 7 Department of Zoology, College of Science, King Saud University, P.O. Box 2455, Riyadh 11451, Saudi Arabia; salkahtani@ksu.edu.sa (S.A.); abdeldaim.m@vet.suez.edu.eg (M.M.A.-D.); 8 Pharmacology Department, Faculty of Veterinary Medicine, Suez Canal University, Ismailia 41522, Egypt

**Keywords:** fibrous crop, copper stress, chelating agent, phytoremediation, oxidative stress

## Abstract

Copper (Cu) is an important micronutrient for a plant’s normal growth and development. However, excess amount of Cu in the soil causes many severe problems in plants—which ultimately affect crop productivity and yield. Moreover, excess of Cu contents causes oxidative damage in the plant tissues by generating excess of reactive oxygen species (ROS). The present experiment was designed to investigate the phytoextraction potential of Cu, morpho-physiological features and biochemical reaction of jute (*Corchorus capsularis* L.) seedlings using ethylenediaminetetraacetic acid (EDTA) of 3 mM under different Cu levels (0 (control), 50 and 100 μM) in a hydroponic nutrient solution (Hoagland). Our results showed that elevated Cu rates (50 and 100 μM) in the nutrient solution significantly reduced plant height, fresh and dry biomass, total chlorophyll content and gaseous exchange attributes in *C. capsularis* seedlings. As the concentration of Cu in the medium increased (50 and 100 μM), the level of malondialdehyde (MDA) and oxidative stress in *C. capsularis* seedlings also increased, which could have been controlled by antioxidant activity in particular plant cells. In addition, rising Cu concentration in the nutrient solution also increased Cu uptake and accumulation in roots and leaves. The addition of EDTA to the nutrient solution significantly alleviated Cu toxicity in *C. capsularis* seedlings, showing a significantly increase in plant growth and biomass. MDA contents was not significantly increased in EDTA-induced plants, suggesting that this treatment was helpful in capturing ROS and thereby reducing ROS in in *C. capsularis* seedlings. EDTA modification with Cu, although the bioaccumulation factor in roots and leaves and translocation factor for the leaves of *C. capsularis* seedlings has significantly increased. These results indicate that *C. capsularis* has considerable potential to cope with Cu stress and is capable of removing a large quantity of Cu from the Cu-contaminated soil while using EDTA is a useful strategy to increase plant growth and biomass with Cu absorption capabilities.

## 1. Introduction

Increased concentrations of heavy metals are seriously threatened by soil pollution [1,2,3,4]. Copper (Cu) is an essential plant micronutrient of various heavy metals requiring normal growth and body development [5,6,7,8]. Cu being a micronutrient, cell wall metabolism, nitrogen fixation, protein synthesis, and many other processes in both physiology and biochemistry play a very important role [9,10,11,12]. However, excess Cu in soil is toxic to plants and can affect plant development, physiological processes such as respiration and photosynthesis, altering DNA structure and endangered plant survival [1,13,14,15,16]. Some key factors in Cu’s high soil concentration are the use of fungicides, bactericides and herbicides in agriculture [2,11]. About 16% of the soil is polluted by heavy metals in China and only about 2.1% of the soil is tainted with Cu [3,17,18]. Cu is an essential micronutrient and also a cofactor of many enzymes such as peroxidase (POD) and superoxidase dismutase (SOD) [4,19,20]. However, toxic Cu levels in the plants increased reactive oxygen species (ROS), such as superoxide radical (O^−^), H_2_O_2_, singlet oxygen (^1^O_2_) and hydroxyl radicals (OH). Antioxidants such as SOD and POD therefore, essential for the scavenge of ROS [21,22,23,24,25]. Cu also causes excessive oxidative damage to plants due to malondialdehyde (MDA) content that induces protein peroxidation and bilayer lipid [26,27,28,29,30,31]. Therefore, in order to prevent plant Cu toxicity, it is very important to reduce or reduce the concentration of Cu in soil to an appropriate level.

Phytoextraction, a green technology allowing removing soil contaminants has become increasingly popular due to fact that it is treated cost-effective and extensively popular technique [32,33,34,35]. It is an environmentally friendly, scientifically accepted and more effective method for the removal of heavy metals or other substances by fast-growing plant species for the absorption of these toxic substances into their harvestable parts [17,24,36]. Heavy metals are extracted from the soil to the roots and transported to the shoots, depending on plant species, soil types, the availability of heavy metals, the stage of growth and the application of fertilizers [11,12,37]. Numerous plant species have been used to accumulate various heavy metals, such as lead (Pb), zinc (Zn), copper (Cu) and cadmium (Cd), etc., [38,39,40]. Different fibrous species such as *Corchorus capsularis*, *Boehmeria nivea* and *Linum usitatissimum* have been used for the phytoextraction of different heavy metals due to effective amount of heavy metals accumulation in their body parts [41,42,43]. In previous studies, *C. capsularis* was used to phytoextract Pb, Zn, Cu and Cd from the soil. Due to the fast growth and enormous biomass increasement and also owing to physiological and biochemical processes, *C. capsularis* is more tolerant to heavy metals than other fibrous crops [42,44,45]. Thus, *C. capsularis* could be an ideal candidate to remove Cu from contaminated soil. The detailed characteristics of *C. capsularis* to accumulate different heavy metals from the metal contaminated soils are previously discussed in a review of literature by Saleem et al. [46].

Chelators such as ethylenediaminetetraacetic acid (EDTA) chelate different heavy metals in the soil. In cause of, most heavy metals with low soil bioavailability and various chelating agents, such as EDTA, have been applied to plants to improve the bioavailability of metals [47,48,49]. Organic chelating agents such as EDTA are more efficient, environmentally friendly and biodegradable compared to inorganic chelating agents. EDTA is a scientifically accepted chelating agent for improving the solubility, absorption and stability of metals. Therefore, EDTA was used in many studies to improve the accumulation and transfer of metal through the soil, as well as to promote the growth and development of plants when grown in metal-polluted soils [50,51]. To enhance the metal uptake in plants, very few amount of chelators are required for example 2-mM EDTA and NTA is enough to increase the Cd uptake in *Sigesbeckia orientalis* [52]. Much research has been published on EDTA-assisted phytoextraction of different heavy metals in many plant species [53,54,55,56], but very few studies have examined the EDTA-assisted Cu *C. capsularis* phytoextraction effect. The study aims to (i) evaluate the potential of EDTA for increasing the phytoextraction of Cu in *C. capsularis* seedlings and (ii) determine its role in promoting morpho-physiological traits and gaseous exchange attributes while alleviating oxidative stress in *C. capsularis* seedlings.

## 2. Materials and Methods

### 2.1. Plant Growth Conditions and Treatments

Mature seeds of *C. capsularis* (C-3) released from Bangladesh, washed with distilled water and sowed in the experimental fields of Huazhong Agricultural University Wuhan, China. The seeds of jute (*Corchorus capsularis*) were collected from Bast and Fiber Research Center, Huazhong Agricultural University, Hubei Province, P.R. China. The same *C. capsularis* type (C-3) is a hyperaccumulator species for Cu contaminated soil which was previously used in many studies [29,57,58,59,60,61]. After two weeks of seed sowing a uniform size of seedlings was collected and converted into a 150 mL flask containing the Hoagland solution nutrient (pH 6.5) and put in germinating machine, where the volumetric flasks were placed in the growth chamber (day/night temperature at 25/20 °C) with 12 h light (13,000 lx) and 12 h dark (HP250GS-C, Ruihua Instrument and Equipment Co., Ltd., Wuhan, Hubei, China) of Huazhong Agricultural University. Plants were able to grow in nutrient solution and nutrient solution was spiked artificially with various levels of Cu using CuSO_4_ 5H_2_O (99% purity) and EDTA as follows: Cu (0 μM/L), Cu (0 μM/L) + EDTA (3 mM), Cu (50 μM/L), Cu (50 μM/L) + EDTA (3 mM) and Cu (100 μM/L), Cu (100 μM/L) + EDTA (3 mM). The experiment was conducted in a completely randomized design (CRD), with one plant in each flask and three replications. The nutrient solution was renewed three times a week to prevent any microbial or fungal attacks. At juvenile stage EDTA was applied as already used previously by Chigbo et al. [62]. In this experiment, we used EDTA concentration (3 mM) which was slightly higher than used by Azhar et al. [63] and Habiba et al. [24]. Nutrient solution pH was maintained throughout the experiment using 1-M H_2_SO_4_ and NaOH at 6.5 ± 0.2. After four weeks of treatment, all plants were wrapped in different plant parts for different characteristics of morphophysiological and metal accumulation in different plant parts. All chemicals used were of analytical grade, procured from Sinopharm Chemical Reagent Co., Ltd.

### 2.2. Sampling and Data Collection

Plants were collected and plant height, diameter, fresh and dry biomass was measured after 28 days of providing EDTA with a nutrient solution. The height of the plant was determined by measuring the scale from the tips of the shoot to the root hair. The diameter of the plant was determined using Vernier (ST22302 SG Devices, Hangzhou, China). Complete fresh weight was determined by calculating the weight of roots and shoots using a weighing balance, and plants were dried for 72 h at 65 °C and dry weight was measured until weight was constant. The leaves have also been collected for the testing of enzymes and washed in liquid nitrogen at −80 °C with distilled water for further analysis. Roots were immersed in 20-mM Na2EDTA for 15–20 min to remove Cu adhered to the surface of roots. Then, roots were washed thrice with distilled water and finally once with deionized water and dried for further analysis [64].

### 2.3. Determination of Chlorophyll Contents and Gaseous Exchange Parameters

Leaves were collected for determination of chlorophyll content. For chlorophyll content analysis, 0.1 g of fresh leaf sample was extracted with 8 mL of 95% acetone for 24 h at 4 °C in the dark. The absorbance was measured by a spectrophotometer (UV-2550; Shimadzu, Kyoto, Japan) at 646.6, 663.6 and 450 nm. Chlorophyll content was calculated by the standard method of Arnon [65].

At the same days, gaseous exchange was also measured. Net photosynthesis (Pn), leaf stomatal conductance (gs), transpiration rate (Ts), and intercellular carbon dioxide concentration (Ci) were measured from three different plants in each treatment group. Measurements were conducted between 11:30 and 13:30 on days with clear sky. Rates of leaf Pn, gs, Ts and Ci were measured with a LI-COR gas-exchange system (LI-6400; LI-COR Biosciences, Lincoln, NE, USA) with a red–blue LED light source on the leaf chamber. In the LI-COR cuvette, CO_2_ concentration was set as 380 mmol mol^−1^ and LED light intensity was set at 1000 mmol m^−2^ s^−1^, which is the average saturation intensity for photosynthesis in *C. capsularis* [66].

### 2.4. Determination of Contents of Malondialdehyde and Proline and Activities of Antioxidant Enzyme

The degree of lipid peroxidation was evaluated as malondialdehyde (MDA) content. Briefly, 0.1 g of frozen leaves were ground at 4 °C in a mortar with 25 mL of 50-mM phosphate buffer solution (pH 7.8) containing 1% polyethylene pyrrole. The homogenate was centrifuged at 10,000× *g* at 4 °C for 15 min. The mixtures were heated at 100 °C for 15–30 min and then quickly cooled in an ice bath. The absorbance of the supernatant was recorded by using a spectrophotometer (xMark™ microplate absorbance spectrophotometer; Bio-Rad, USA) at wavelengths of 532, 600 and 450 nm. Lipid peroxidation was expressed as l mol g^−1^ using the following formula: 6.45 × (A532−A600) − 0.56 × A450. Lipid peroxidation was measured using a method previously published by Health and Packer [67]. Proline contents were determined by the method of Bates et al. [68] using a standard curve prepared with proline.

To evaluate enzyme activities, fresh leaves (0.5 g) were homogenized in liquid nitrogen and 5 mL of 50-mmol sodium phosphate buffer (pH 7.0) including 0.5-mmol EDTA and 0.15-mol NaCl. The homogenate was centrifuged at 12,000× *g* for 10 min at 4 °C, and the supernatant was used for measurement of SOD and POD activities. SOD activity was assayed in 3 mL reaction mixture containing 50-mM sodium phosphate buffer (pH 7), 56-mM nitro-blue tetrazolium, 1.17-mM riboflavin, 10-mM methionine and 100 μL enzyme extract. Finally, the sample was measured by using spectrophotometer (xMark™ microplate absorbance spectrophotometer; Bio-Rad). Enzyme activity was measured using a method by Chen and Pan [69], and expressed as U g^−1^ FW.

POD activity in the leaves was estimated using the method of Sakharov and Ardila [69] using guaiacol as the substrate. A reaction mixture (3 mL) containing 0.05 mL of enzyme extract, 2.75 mL of 50-mM phosphate buffer (pH 7.0), 0.1 mL of 1% H_2_O_2_ and 0.1 mL of 4% guaiacol solution was prepared. Increases in the absorbance at 470 nm due to guaiacol oxidation was recorded for 2 min. One unit of enzyme activity was defined as the amount of the enzyme.

### 2.5. Cu Determination

Dried root and shoot samples were ground in a stainless-steel mill and passed through a 0.1-mm nylon sieve for Cu analysis. Briefly, 0.1 g of dried sample was digested in HNO_3_/HClO_4_ (4:1) solution. Digested solution was washed in 25-mL flasks and diluted in deionized water until reaching the final volume of 25-mL. The supernatant was passed through a 0.45-μm filter paper and analyzed for Cu content by an atomic absorption spectrophotometer (240FS-AA; Agilent).

Bioaccumulation factor (BAF) was calculated as the ratio of Cu concentration in tissues and Cu concentration in the nutrient solution by using the following formula:

(1) $$\mathrm{BAF}=\frac{\mathrm{Cu}\;\mathrm{concentration}\;\mathrm{in}\;\mathrm{plant}\;\mathrm{tissues}}{\mathrm{Cu}\;\mathrm{concentration}\;\mathrm{in}\;\mathrm{the}\;\mathrm{nutrient}\;\mathrm{solution}}$$

while translocation factor (TF) was determined by estimating the concentration of Cu in one part of plant with respect to the other parts as follows:

(2) $$\mathrm{TF}=\frac{\mathrm{Cu}\;\mathrm{concentration}\;\mathrm{in}\;\mathrm{plant}\;\mathrm{tissues}}{\mathrm{Cu}\;\mathrm{concentration}\;\mathrm{in}\;\mathrm{roots}}$$

### 2.6. Statistical Analysis

Standard deviation (SD) were considered significant when the *P*-values were less than 0.05 after comparison with Tukey’s HSD post hoc test. The important treatments used in this study were evaluated by the regression analysis using Statistix 8.1. The one-way analysis of variance (ANOVA) was used to analyze differences in different morphologic and physiological traits. Standard errors were considered significant when the *P*-values were less than 0.05 after comparison with Tukey’s HSD post hoc test. Graphical presentation was carried out using SigmaPlot 12.

## 3. Results

### 3.1. Plant Growth and Biomass

Plant growth and biomass were considerably reduced in compared to untreated EDTA in terms of plant height, plant diameter, fresh and dry bio-mass by adding higher Cu concentrations (50 and 10 μmol/L) in the nutrient solution (*P* < 0.05) (Table 1). Maximum plant height, plant diameter, plant fresh weight and plant dry weight reduction were measured at highest Cu treatment, i.e., 100 μmol/L which caused 52%, 25%, 22% and 35%, respectively as compared with control. While at 50 μmol/L, the following traits were also decreased and decreased by 35%, 10%, 8% and 17% compared to the control treatment. The application of EDTA in the nutrient solution of Cu contaminated mixture of *C. capsularis* significantly (*P* < 0.05) revoke metal toxicity by improvement in plant growth and biomass production. The results revealed that Cu-stressed plants, i.e., 100 μmol/L with the application of EDTA, i.e., 3 mM exhibited 9%, 7%, 6% and 11% increase in plant height, plant diameter, fresh weight and dry weight by, respectively, when compared with 100 μmol/L without the application of EDTA.

### 3.2. Chlorophyll Contents and Gaseous Exchange Attributes

Results regarding with different levels of Cu (0, 50 and 100 μmol/L) with or without the application of EDTA on total chlorophyll contents of *C. capsularis* are presented in Table 1. According to the results, it was observed that increasing level of Cu (50 and 100 μmol/L) in the nutrient solution significantly decreased the total contents of chlorophyll in the leaves of *C. capsularis*. However, application of EDTA to the Cu-stressed plants significantly (*P* < 0.05) improved photosynthetic pigments in *C. capsularis*. The increase in total chlorophyll contents was 21% in 100 μmol/L with EDTA compared to the corresponding treatment without EDTA use.

**Figure 1:**
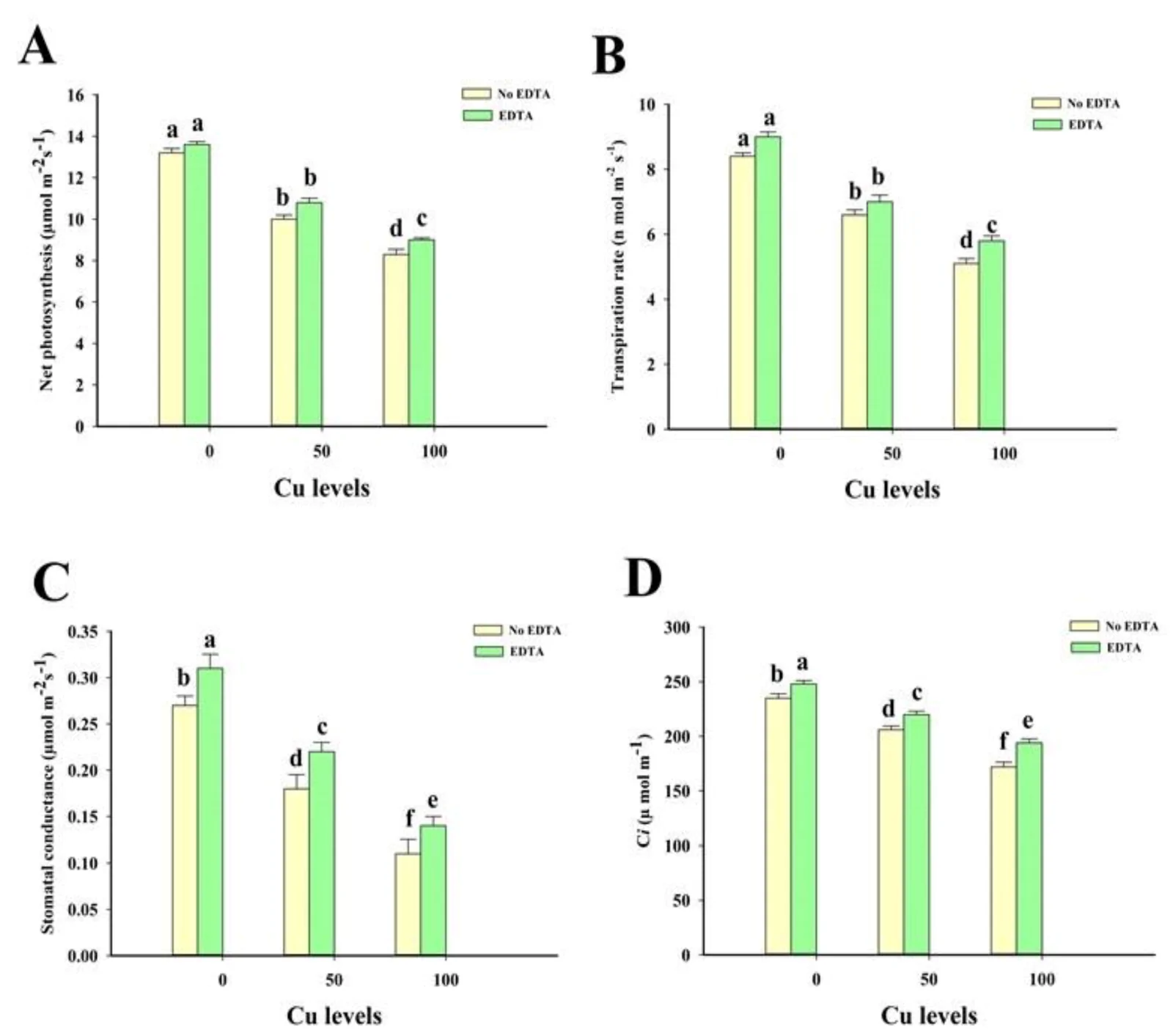
Effect of different concentrations of Cu with or without the application of EDTA on net photosynthesis (**A**), transpiration rate (**B**), stomatal conductance (**C**) and intercellular CO_2_ (**D**) in the leaves of *C. capsularis* seedlings grown in solution medium with increasing Cu concentrations (0, 50 and 100 μM) treated with and without 3-mM EDTA. The given values are means ± SD (n = 3). One-way ANOVA was performed and means differences were tested by highest significant difference HSD (*P* < 0.05). Different lowercase letters in figure indicate significant difference between the treatments.

In the present study, increasing concentration of Cu in the nutrient solution (50, 100 μmol/L) significantly decreased P*n*, T*r*, G*s* and C*i* of *C. capsularis* seedlings (Figure 1). These results suggested that the application of EDTA to Cu treated plants significantly increased gaseous exchange attributes compared with the plants grown under Cu-only treatment. The application of EDTA to plants treated with 50 μmol/L induces 8%, 6%, 22% and 7% increase in P*n*, T*r*, G*s* and C*i,* respectively compared to plants grown under 50 μmol/L without EDTA application. In the same way, the plants grown under 100 μmol/L treated with EDTA significantly increased P*n*, T*r*, G*s* and C*i,* respectively by 8%, 14%, 27% and 13% compared with the plants grown under 100-μmol/L without the application of EDTA.

### 3.3. Oxidative Stress and Antioxidant Enzyme Activities

**Figure 2:**
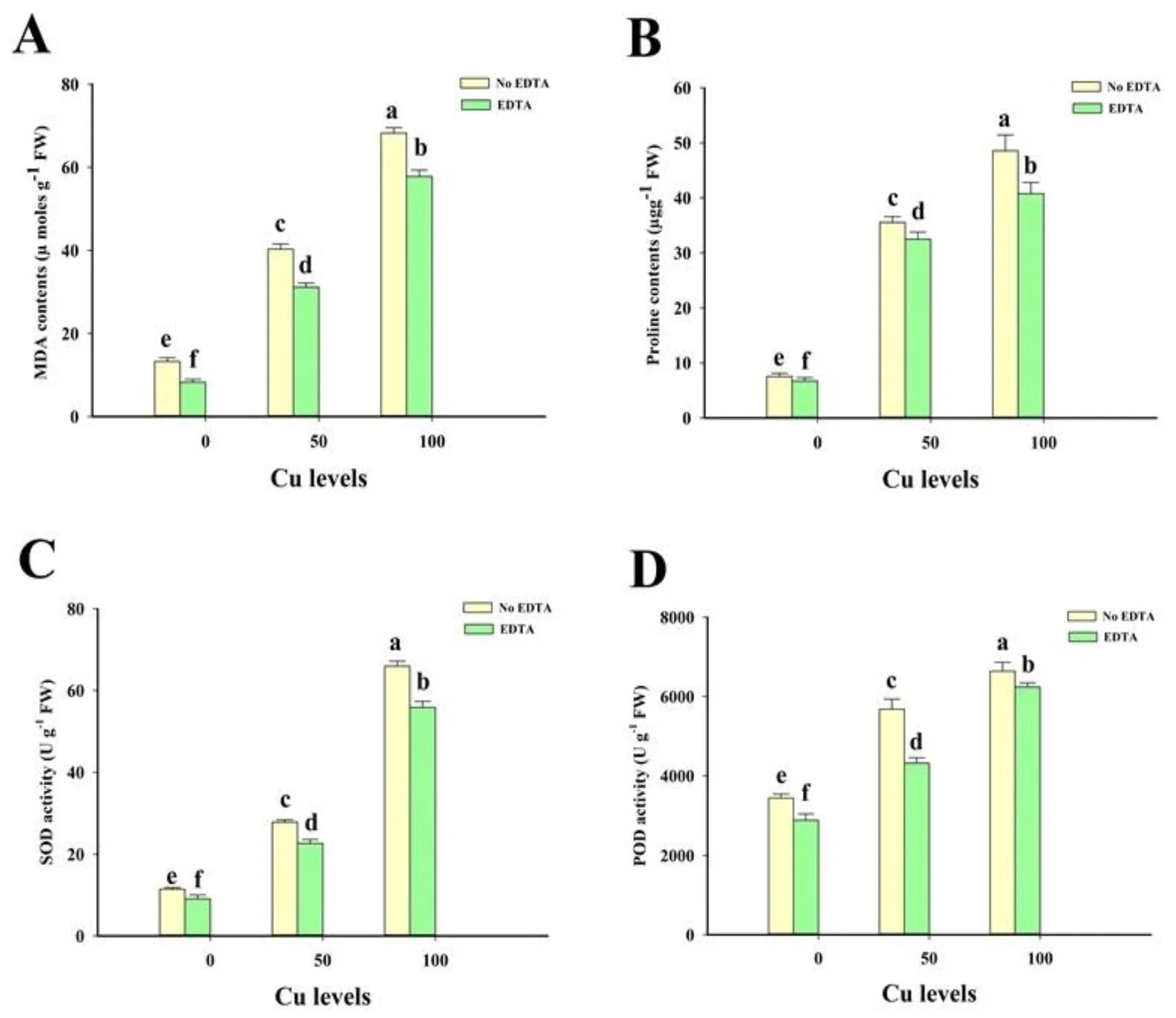
Effect of different concentrations of Cu with or without the application of EDTA on malondialdehyde contents (**A**), proline contents (**B**), superoxidase activity (**C**) and peroxidase activity (**D**) in the roots of *C. capsularis* seedlings grown in solution medium with increasing Cu concentrations (0, 50 and 100 μM) treated with and without 3-mM EDTA. The given values are means ± SD (n = 3). One-way ANOVA was performed and means differences were tested by highest significant difference HSD (*P* < 0.05). Different lowercase letters in figure indicate significant difference between the treatments.

**Figure 3:**
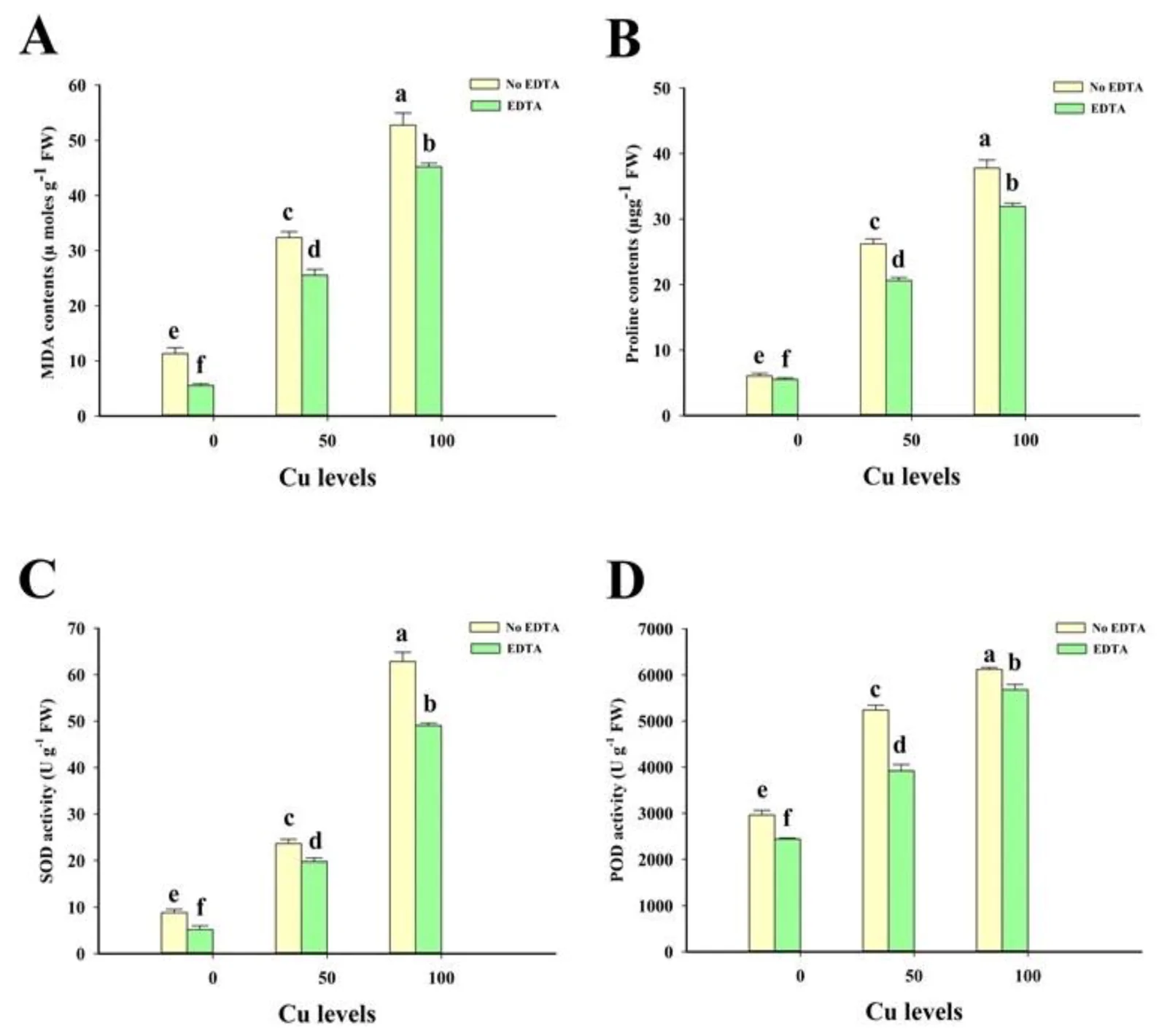
Effect of different concentrations of Cu with or without the application of EDTA on malondialdehyde contents (**A**), proline contents (**B**), superoxidase activity (**C**) and peroxidase activity (**D**) in the leaves of *C. capsularis* seedlings grown in solution medium with increasing Cu concentrations (0, 50 and 100 μM) treated with and without 3-mM EDTA. The given values are means ± SD (n = 3). One-way ANOVA was performed and means differences were tested by highest significant difference HSD (*P* < 0.05). Different lowercase letters in figure indicate significant difference between the treatments.

In this research, the influence of different levels of Cu (50 and 100 μmol/L) and application of EDTA (3 mM) on lipid peroxidation (MDA content), proline content and antioxidant enzyme activity (SOD and POD) in *C. capsularis* roots and leaves were also examined (Figure 2, Figure 3). The use of EDTA, however, reduces MDA, proline contents and SOD and POD activities in *C. capsularis* roots and leaves. Moreover, increasing contents of MDA contents suggested that Cu toxicity induced oxidative damage in *C. capsularis*. Compared to the control treatment, the overall increase according to the results in the roots and leaves of 475% and 372%, respectively in MDA contents were recorded at Cu100 followed by Cu100 + EDTA (338% and 309%, respectively) and Cu50 (207% and 190%, respectively) than the treatment without Cu and EDTA. However, the minimum MDA contents in the roots and leaves were found in the treatments where EDTA is applied without Cu level (8 and 6 µmoles g^−1^ FW, respectively) followed by control (13 and 11 µmoles g^−1^ FW. The proline in the leaves has substantially been increased (*P* < 0.05), while EDTA has decreased the proline in the roots and leaves of the *C. capsularis* significantly (*P* < 0.05) with Cu in the nutrient solution. The maximum increase in the roots and leaves of 540% and 533%, respectively in proline contents were observed in Cu100 followed by Cu100 + EDTA (446% and 433%) and Cu50 (400% and 336%) than the treatment without Cu and EDTA. However, the minimum proline contents in the roots and leaves were found in the treatments where only EDTA was applied (7 and 6 µgg^−1^ FW) followed by control (8 and 7 µgg^−1^ FW).

Increased Cu levels in the nutrient solution (50 and 100 μmol/L) were found to significantly enhance the activities of various enzymatic antioxidants such as SOD and POD in the roots and leaves of *C. capsularis* seedlings. However, exogenous supplementation of EDTA decreased the activities of antioxidant enzymes (Figure 2, Figure 3). SOD was observed at Cu100 in the roots and leaves (66 and 63 U g^−1^ FW, respectively) and PODs (6640 and 6120 U g^−1^ FW, respectively), followed by Cu100 + EDTA in the roots and leaves in the leaves of *C. capsularis* (6240 and 5680 Ugg^−1^ FW, respectively). Using EDTA (3 mM) significantly reduced SOD and POD enzymatic activity compared to plants grown without EDTA use. Nonetheless, the minimum value in SOD roots and leaves was observed in plants grown using EDTA without Cu (9 and 5 U g^−1^ FW, respectively) and POD (2880 and 2440 U g^−1^ FW, respectively) compared to the control.

### 3.4. Uptake and Distribution of Cu

In this study, determination of Cu concentration in different parts of *C. Capsularis* (roots and leaves) seedlings under various levels of Cu (50 and 100 μmol/L) with or without the application of EDTA (3 mM) were also studied. However, the determination of Cu concentration from different parts of the plants were measured after 28 days of given the treatments to *C. capsularis* seedlings (Table 2). These results suggested that significantly increased Cu concentration in the nutrient solution (*P* < 0.05) caused an increase in Cu contents in the roots and leaves of *C. capsularis*. It was also noticed that application of EDTA to the Cu-stressed plants also helps in the Cu accumulation in *C. capsularis*. Results also show that the highest concentration of Cu was observed in the roots (57 mg/kg Cu) while few transported to the leaves (45 mg/kg Cu) compared to regulation at Cu100 + EDTA. The maximum Cu concentration was observed in the roots at Cu100 + EDTA (57 mg/kg Cu), followed by Cu100 (49 mg/kg Cu) and Cu50 + EDTA (37 mg/kg Cu) compared to the display. Similarly, Cu100 + EDTA (45 mg/kg Cu) was observed in the leaves, followed by Cu100 (40 mg/kg Cu) and Cu50 + EDTA (33 mg/kg Cu) compared to control. These findings showed that application of EDTA enhances the Cu accumulation as follows: Cu100 + EDTA > Cu100 > Cu50 + EDTA > Cu50 > EDTA > control.

**Figure 4:**
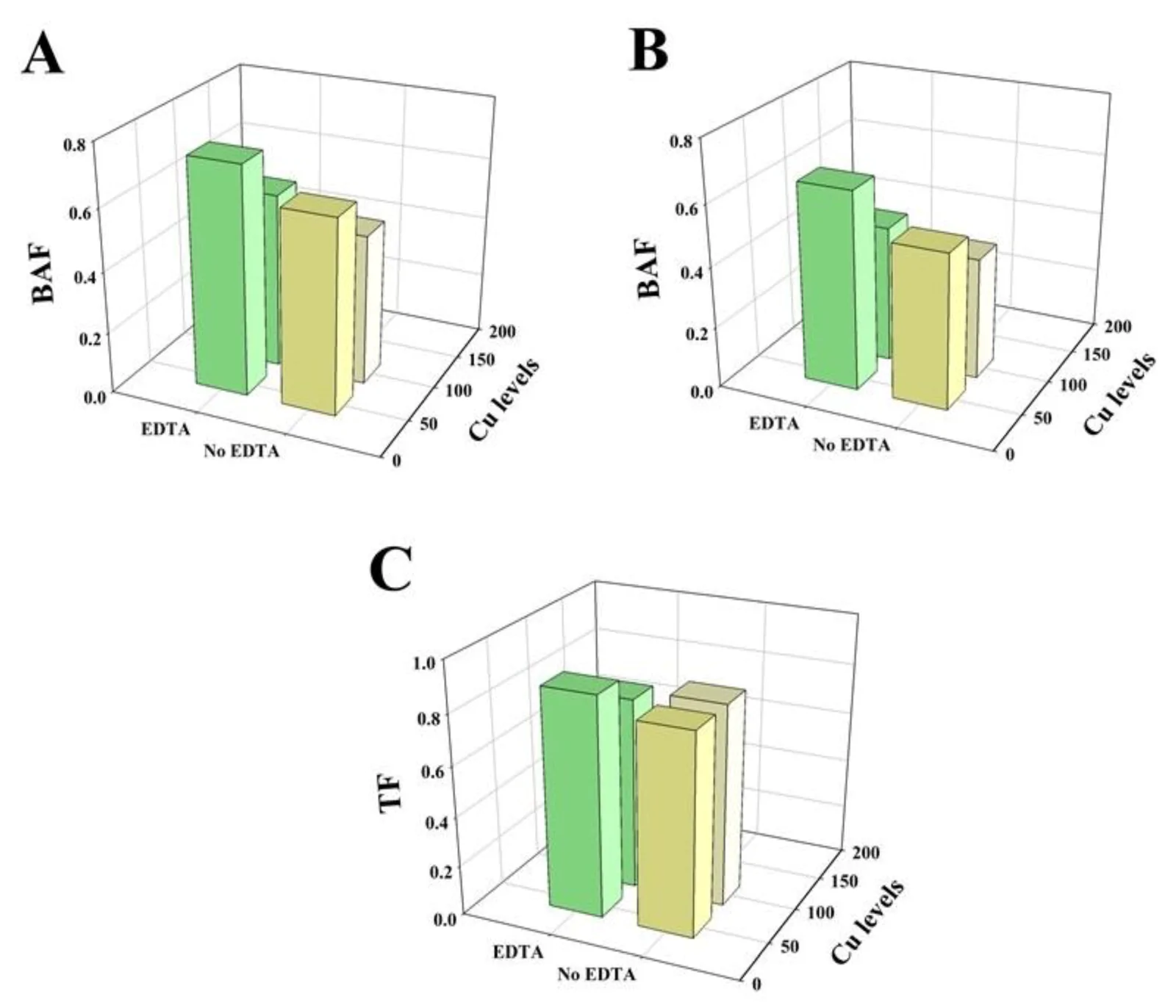
Effect of different concentrations of Cu and with or without the application of EDTA on bioaccumulation factor in roots (**A**), bioaccumulation factor in shoots (**B**) and translocation factor in the shoots (**C**) of *C. capsularis* seedlings grown in solution mixture with increasing Cu concentrations (0-, 50- and 100-μM) treated with and without 3-mM EDTA.

BAF and TF in *C. Capsularis* seedlings are presented in Figure 4. It was noted that all BAF and TF values are less than 1 while applying EDTA to Cu-stressed plants showed higher BAF and TF values compared to plants without EDTA. The minimum value of TF was observed in Cu100 + EDTA (0.77) while maximum TF value was observed in Cu50 + EDTA (0.88). The highest BAF value was recorded at Cu50 + EDTA (0.74) in the roots while (0.65) in the leaves. The values of BAF and TF were increased with the application of EDTA to the Cu stressed plants when grown in the nutrient solution medium.

## 4. Discussion

Results from this study suggested that increased concentrations of Cu (50 and 100 μmol/L) in nutrient solution (*P* < 0.05) significantly decrease plant growth and biomass relative to control (Table 1). High concentration of Cu in growth medium significantly reduced plant growth and biomass reported in many previous studies [5,9,70,71,72,73,74]. The decrease in plant growth and biomass under high Cu concentration in the nutrient solution may be due to high Cu accumulation in different parts of *C. capsularis* (Table 2) triggering phytotoxic effects [16,75,76,77]. The toxicity of elevated levels of metal ions upon the pH, composition and concentration of medium and species [7,11,78]. The toxic Cu concentration in the medium can cause nutrient imbalance by binding with iron and manganese oxides that affect the productivity of plants and crop yield. The destabilization of plant growth and development in high concentrations of Cu are commonly observed reactions [5,13,74,79,80].

With the rise in Cu concentration in the nutrient solution, total chlorophyll content and gaseous exchange attributes were also reduced (Table 1, Figure 1). Increasing level of Cu concentration in the medium significantly decrease photosynthesis in the leaves. However, the main effect of Cu concentration is inhibition of electron transport chain, reduce photosynthesis rate [75,81]. Ahmed and Slima [82] reported that the significant decrease in chlorophyll contents in *C. capsularis* when subjected to different heavy metals such as Pb, Cu, Cr, Fe and Zn. The decrease in chlorophyll contents under Cu stress has been studied previously [15,24,83,84] while reduction in gaseous exchange attributes under Cd stress has been studied by Ali et al. [85]. The drop in chlorophyll in the leaves of *C. capsularis* may be due to the displacement of Mg ion which is required for synthesis of chlorophyll [7,24,86].

Cu in excess also involves in the generation of large amount of reactive oxygen species (ROS) because of depletion of low molecular antioxidants and distribution of metabolic pathways that shows oxidative stress in plants [5,13,21,87,88,89]. ROS production in the leaves is scavenging by antioxidants such as SOD and POD [16,17,90,91,92,93]. In this analysis, high concentration of Cu in the nutrient solution causes high MDA content, proline, and increased enzymes with antioxidant activities such as SOD and POD in *C. capsularis* roots and leaves (Figure 2, Figure 3). These results are in agreements with previous studies using Cu as heavy metals [24,26,85,90,94]. It was also reported that excess Cu increases lipid peroxidation indicating that prevalence in membrane bounded organelles [5,16,28]. In addition, oxidative stress rises in *C. capsularis* roots and leaves. The increase of Cu in a nutrient solution which is a stress factor that causes impaired plant growth and development due to Cu toxicity (Figure 2). The contents of proline in the plant tissues are beneficial in signal transduction associated with Cu toxicity and avoids distortion of the membrane due to oxidative stress. Similar results were showed by [10] when they studied *Boehmeria nivea* under different levels of Cu and noticed that proline contents in the leaves were continuously increased with the increase in Cu concentration in the soil. To decrease the oxidative stress in the plants, plants have developed effective antioxidants such as (SOD and POD). In the present study, the enzymatic activities of SOD and POD in the roots and leaves of *C. capsularis* were also increased with the increase in Cu concentration in the nutrient solution (Figure 2C,D). The process of ROS detoxification in the plants is dispensable by the activation of these antioxidants which ultimately scavenge these species (ROS) [16,22,73,95]. The increase in antioxidants under Cu-stressed plants were observed in *Boehmeria nivea* [43] and *Oryza sativa* [16]. However, continuously increasing in the activities of SOD and POD under high concentration of Cu indicating that *C. capsularis* can tolerate better than other many plant species. Moreover, the persistent increase in antioxidant activity may be due to changes in gene expression and protein function [91,96,97]. However, better growth and development of a plant under low concentration of Cu is also associated with active antioxidant defense system [4,17,98,99,100].

The uptake and translocation of heavy metals in the plants grown under metal contaminated soil mainly depends upon the metal supply and growth conditions [10,12,54,101]. Results of this study indicated that Cu concentration in roots and leaves increased as Cu concentration in the nutrient solution increased (Table 2). The highest concentration of Cu in the roots and leaves was estimated at 100 μmol/L, i.e., 49 and 40 mg/kg, respectively, and Cu was also found to be highly accumulated in the roots while being transported little to the harvestable parts of the plant (Table 2). These findings are agreement with the findings of Bhattacharya et al. and Niazy and Wahdan [42,45] whom found a high concentration of Cu, Co, Ni and Fe in the *C. capsularis* and was transported to the stems and leaves. One more possible reason behind this is that the time period of Cu treatment was only 28 days. Bhattacharya et al. [42] studied the mechanism behind this and reported that in early stage of *C. capsularis* iron plaque in formed in the roots and inhibits the transportation of As to the aboveground parts of the plants. However, in the lateral stage of the growth As was highly transported to the above ground parts with little accumulation in the roots. It is also believed that the restriction of the plants to uptake/accumulate different metal contents in their aboveground parts of the plant from the roots can be considered as a tolerance mechanism of a plant against metal stress [77,102,103]. However, the maximum concentration of Cu was accumulated in the roots with little transported to the shoots; this is why the values of TF were less than 1 (Figure 3). The highest value of BAF in the roots and leaves were 0.63 and 0.51, respectively, while highest value of TF was 0.81 recorded at Cu50. These findings are similar to the findings of Ahmed and Slima [82], who studied different heavy metals and noticed that under high concentration of Cd and Ni the values of BAF and TF in *C. capsularis* were less than 1. Yoon et al. [104] and Chen et al. [17] studied *Gentiana pennelliana* and *Moso bamboo,* respectively, and noticed that Cu strongly accumulated in the roots, while little was transported into the shoots—rendering both BAF and TF values less than one in all treatments. In our previous study, we noticed that high concentrations of Cu in the soil (a pot experiment) destroyed the cellular structure of *C. capsularis* plants due to high metal toxicity that directly affected photosynthetic machinery [58].

EDTA significantly increased plant growth and biomass compared to Cu-stressed plants (Table 1). Application of EDTA increased plant growth and development in *Dianthus chinensis*, *Chlorophytum comosum* and *Zea mays* [15,105]. The increase in plant growth and biomass using EDTA in Cu-stressed plants may be due to the enhanced gaseous exchange attributes using EDTA (Figure 1). Another possible reason is the formation of chelate with the Cu^2+^ that may reduce the toxic effects of Cu [24,47,48]. Although the application of EDTA is independent of metal stress as it increased plant growth and biomass (even under normal conditions), this may be due to increased nutrient uptake and/or EDTA induced chelation of metals decreasing free metal ions in plants, as suggested by Kanwal et al. [56]. In the present study, chlorophyll contents and gaseous exchange attributes were also increased by the application of EDTA, as compared to plants grown without application of EDTA (Table 1, Figure 1). The conversion of light energy into photochemical reactions is more efficient under the application of EDTA in Cu-stressed plants may also play a key role in improving growth and development [49,106]. Increased photosynthetic pigments and gaseous exchange attributes under EDTA agree with Kanwal et al. [56] who reported that application of EDTA under lead-stressed-*Brassica napus* plants. Kanwal et al. [56] also reported that application of EDTA reduced oxidative stress under high concentration of lead as indicated by decrease in MDA contents which showed similar trends with our study (Figure 2). Moreover, increase in photosynthetic rate may be due to the protective role of EDTA on photosynthetic machinery by reducing the metal free ions and increase the activities of antioxidants which ultimately reduced oxidative stress in *C. capsularis* [53,107]. The application of EDTA can form chelates with Cu^2+^ and can reduced oxidative stress generated by Cu toxicity (Figure 2). The increase in the antioxidative enzymes (SOD and POD) known as mediators for oxidative stress in plants may protect a plant from the toxic effects of ROS. Moreover, increasing activities of SOD and POD could increase the ability of a plant to scavenge ROS and prevent oxidative stress in plants [53,108]. In the present experiment, increasing growth, biomass, gaseous exchange attributes and alleviates oxidative stress is directly linked with the activities of antioxidants (Figure 2) with the application of EDTA under elevating levels of Cu in the nutrient solution.

The application of EDTA further increase the concentration of Cu in the roots and leaves of *C. capsularis* under different levels of Cu in nutrient solution (Table 2). It was also noticed that the values of BAF and TF were also increased with the application of EDTA compared with the plants without the application of EDTA under Cu treated plants (Figure 3). The increase in Cu uptake with the application of EDTA in Cu-stressed plants may be due to chelating of EDTA with Cu [55,56,109,110]. However, increase in growth and biomass with the application of EDTA in Cu-stressed plants is suggesting that *C. capsularis* is tolerant to Cu stress with active antioxidative defense system (Figure 2). The application of EDTA increase metals availability has been reported in many previous studies [24,47,51,56,111]. One more possible reason behind this mechanism is that roots are capable of liberating trace metals from dissociated organometallic compounds and this process increase metal uptake in the plants [24,84,112].

## 5. Conclusions

Based on these results, high concentrations of Cu in the nutrient solution decreased plant growth, biomass, chlorophyll contents and gaseous exchange attributes, while inducing oxidative damage by generated high amount of ROS in the tissues of the plant. The negative impact of Cu toxicity in *C. capsularis* can be overcome by the application of EDTA, which increases plant growth, biomass and chlorophyll by ameliorating the oxidative stress (MDA contents) generated by Cu toxicity due to capturing free oxide ions and/or Cu-chelation. Moreover, application of EDTA also assisted phytoextraction of Cu in *C. capsularis* and can regulate plant growth and development and may be a green alternation to conventionally costly and not environment friendly physical–chemical technologies.

## Figures and Tables

**Table 1:** Effect of different concentrations of Cu and with or without the application of EDTA on plant height (cm), plant diameter (mm), plant fresh weight (g), plant dry weight (g) and total chlorophyll contents (mg/g fresh weight) of *C. capsularis* seedlings.

Treatments	Plant Height	Plant Diameter	Plant Fresh Weight	Plant Dry Weight	Total Chlorophyll
Control	23 ± 0.2 a	2.0 1± 0.03 b	2.43 ± 0.04 b	1.59 ± 0.03 b	3 ± 0.05 b
EDTA	23 ± 0.3 a	2.09 ± 0.01 a	2.58 ± 0.02 a	1.65 ± 0.03 a	3.2 ± 0.09 a
Cu50	15 ± 0.1 c	1.79 ± 0.04 d	2.25 ± 0.04 d	1.33 ± 0.02 d	2 ± 0.04 d
Cu50 + EDTA	16 ± 0.2 b	1.89 ± 0.02 c	2.35 ± 0.03 c	1.43 ± 0.02 c	2.1 ± 0.06 c
Cu100	11 ± 0.2 e	1.51 ± 0.03 f	1.89 ± 0.05 f	1.03 ± 0.02 f	1.4 ± 0.09 f
Cu100 + EDTA	12 ± 0.3 d	1.62 ± 0.02 e	2.01 ± 0.05 e	1.14 ± 0.03 e	1.7 ± 0.03 e

The given values are means ± SD (n = 3). One-way ANOVA was performed and means differences were tested by highest significant difference HSD (*P* < 0.05). Different lowercase letters in table indicate significant difference between the treatments. Different abbreviations are used are as follows: control (0-μmol/L Cu + 0-mM EDTA), EDTA (0-μmol/L Cu + 3-mM EDTA), Cu50 (50-μmol/L Cu + 0-mM EDTA), Cu50 + EDTA (50-μmol/L Cu + 3-mM EDTA), Cu100 (100-μmol/L Cu + 0-mM EDTA) and Cu100 + EDTA (100-μmol/L Cu + 3-mM EDTA).

**Table 2:** Effect of different concentrations of Cu and with or without the application of EDTA on Cu accumulation in roots (mg/g FW) and shoots (mg/g FW) of *C. capsularis* seedlings.

Treatments	Cu Concentration in Roots	Cu Concentration in Shoots
Control	13 ± 2.5 e	10 ± 1.4 f
EDTA	15 ± 2.5 e	15 ± 1.4 f
Cu50	32 ± 0.5 d	26 ± 1 d
Cu50 + EDTA	37 ± 1.2 c	33 ± 0.9 c
Cu100	49 ± 1 b	40 ± 0.8 b
Cu100 + EDTA	57 ± 1 a	45 ± 1 a

Values are means ± SD (n = 3). One-way ANOVA was performed and means differences were tested by highest significant difference HSD (*P* < 0.05). Different lowercase letters in table indicate significant difference between the treatments. Different abbreviations are used are as follows: control (0-μmol/L Cu + 0-mM EDTA), EDTA (0-μmol/L Cu + 3-mM EDTA), Cu50 (50-μmol/L Cu + 0-mM EDTA), Cu50 + EDTA (50-μmol/L Cu + 3-mM EDTA), Cu100 (100-μmol/L Cu + 0-mM EDTA) and Cu100 + EDTA (100-μmol/L Cu + 3-mM EDTA).
