# Correction: Saleem et al. Ethylenediaminetetraacetic Acid (EDTA) Mitigates the Toxic Effect of Excessive Copper Concentrations on Growth and Gaseous Exchange of *Corchorus capsularis* L. and Improves Copper Accumulation Capabilities. *Plants* 2020, *9*, 756

**DOI:** 10.3390/plants15192981

**Published:** 2026-09-30

**Authors:** Muhammad Hamzah Saleem, Shafaqat Ali, Muhammad Kamran, Naeem Iqbal, Muhammad Azeem, Muhammad Tariq Javed, Qasim Ali, Muhammad Zulqurnain Haider, Sana Irshad, Muhammad Rizwan, Saad Alkahtani, Mohamed M. Abdel-Daim

**Affiliations:** 1 MOA Key Laboratory of Crop Ecophysiology and Farming System Core in the Middle Reaches of the Yangtze River, College of Plant Science and Technology, Huazhong Agricultural University, Wuhan 430070, China; 2 Department of Environmental Sciences and Engineering, Government College University, Allama Iqbal Road, Faisalabad 38000, Pakistan; mrizwan@gcuf.edu.pk; 3 Department of Biological Sciences and Technology, China Medical University, Taichung 40402, Taiwan; 4 College of Resources and Environment, Huazhong Agricultural University, Wuhan 430070, China; kamiagrarian763@gmail.com; 5 Department of Botany, Government College University, Faisalabad 38000, Pakistan; drnaeem@gcuf.edu.pk (N.I.); mazeem@gcuf.edu.pk (M.A.); mtariqjaved@gcuf.edu.pk (M.T.J.); drqasimali@gcuf.edu.pk (Q.A.); drmzhaider@gcuf.edu.pk (M.Z.H.); 6 School of Environmental Studies, China University of Geosciences, Wuhan 430070, China; sanairshad55@gmail.com; 7 Department of Zoology, College of Science, King Saud University, P.O. Box 2455, Riyadh 11451, Saudi Arabia; salkahtani@ksu.edu.sa (S.A.); abdeldaim.m@vet.suez.edu.eg (M.M.A.-D.); 8 Pharmacology Department, Faculty of Veterinary Medicine, Suez Canal University, Ismailia 41522, Egypt

In the original publication [1], the number of experimental replicates was incorrectly stated as “six replications” in Section 2.1, whereas the tables, figure legends, raw data, and statistical analyses were based on three biological replicates (*n* = 3). In addition, the phrase “Tukey Post hoc (LSD)” in the Statistical Analysis Section was incorrect. The manuscript has been revised as follows:

Section 2.1. Plant Growth Conditions and Treatments: “The experiment was conducted in a completely randomized design (CRD), with one plant in each flask and three replications.”

Section 2.6. Statistical Analysis (renumbered after removal of the former TEM subsection): “Standard deviation (SD) were considered significant when the *P*-values were less than 0.05 after comparison with Tukey’s HSD post hoc test. The important treatments used in this study were evaluated by the regression analysis using Statistix 8.1. The one-way analysis of variance (ANOVA) was used to analyze differences in different morphologic and physiological traits. Standard errors were considered significant when the *P*-values were less than 0.05 after comparison with Tukey’s HSD post hoc test. Graphical presentation was carried out using SigmaPlot 12.”

Figure 5 (Transmission Electron Microscopy experiment) has been removed from the publication. The former Section 2.6, “Transmission Electron Microscopy”; the former Section 3.5, “Transmission Electron Microscopy”; the Figure 5 image and legend; and all statements reporting on or interpreting this experiment have been deleted. The Statistical Analysis subsection has consequently been renumbered as Section 2.6.

To maintain consistency throughout the article, the title, Abstract, Keywords, Introduction, and Discussion have been revised to remove references to the deleted TEM experiment. The corrected title is as follows:

“Ethylenediaminetetraacetic Acid (EDTA) Mitigates the Toxic Effect of Excessive Copper Concentrations on Growth and Gaseous Exchange of *Corchorus capsularis* L. and Improves Copper Accumulation Capabilities”.

The sentence “as well as affected the ultrastructure of chloroplast of *C. capsularis* seedlings” has been removed from the Abstract.

The corrected Keywords are: fibrous crop; copper stress; chelating agent; phytoremediation; oxidative stress.

The final sentence of the Introduction has been revised to state that the study evaluates “The study aims to (i) evaluate the potential of EDTA for increasing the phytoextraction of Cu in *C. capsularis* seedlings and (ii) determine its role in promoting morpho-physiological traits and gaseous exchange attributes while alleviating oxidative stress in *C. capsularis* seedlings.”

All Discussion statements specifically referring to the removed TEM experiment, Figure 5, chloroplast-ultrastructure observations, or TEM-based interpretation have been deleted from the revised manuscript.

The authors state that these corrections do not affect the quantitative results, main findings, or conclusions based on plant growth, gaseous exchange, oxidative-stress and antioxidant responses, and Cu uptake and accumulation. This correction was approved by the Academic Editor. The original publication has also been updated.
